# A country-wide malaria survey in Mozambique. II. Malaria attributable proportion of fever and establishment of malaria case definition in children across different epidemiological settings

**DOI:** 10.1186/1475-2875-8-74

**Published:** 2009-04-21

**Authors:** Samuel Mabunda, John J Aponte, Armindo Tiago, Pedro Alonso

**Affiliations:** 1 National Malaria Control Programme, Maputo, Mozambique; 2 National Institute of Health, Maputo, Mozambique; 3 Centre for International Health, Hospital Clinic, Institut d'Investigacions Biomedicas August Pi i Sunyer (IDIBAPS), University of Barcelona, Barcelona, Spain; 4 Centro de Investigação em Saúde da Manhiça, Maputo, Mozambique; 5 Faculdade de Medicina, Departamento de Fisiologia, Universidade Eduardo Mondlane, Maputo, Mozambique

## Abstract

**Background:**

Protection against clinical malaria episodes is acquired slowly after frequent exposure to malaria parasites. This is reflected by a decrease with increasing age in both parasite density and incidence of clinical episodes. In many settings of stable malaria transmission, the presence of asymptomatic malaria parasite carriers is common and the definition of clinical malaria remains uncertain.

**Methods:**

Between February 2002 and April 2003, a country-wide malaria survey was conducted in 24 districts of Mozambique, aiming to characterize the malaria transmission intensities and to estimate the proportion of fever cases attributable to malaria infections in order to establish the malaria case definition. A total of 8,816 children less than ten years of age were selected for the study. Axillary temperature was measured in all participating subjects and finger prick blood collections were taken to prepare thick and thin films for identification of parasite species and determination of parasite density. The proportion of fever cases attributable to malaria infection was estimated using a logistic regression of the fever on a monotonic function of the parasite density and, using bootstrap facilities, bootstrapped estimated confidence intervals, as well as the sensitivity and specificity for different parasite density cut-offs were produced.

**Results:**

Overall, the prevalence of *Plasmodium falciparum *was 52.4% (4,616/8,816). The prevalence of fever (axillary temperature ≥ 37.5°C) was 9.4% (766/8,816). Fever episodes peaked among children below 12 months of life [15.1% (206/1,517)]. The lowest fever prevalence of 5.9% (67/1,224) was recorded amongst children between five and seven years of age. Among 4,098 parasitized children, 498/4,098 (13.02%) had fever. The prevalence of malaria infections associated with fever peaked among children in the less than twelve months age group and thereafter decreased rapidly with increasing age (p < 0.001). High parasite densities were significantly associated with fever (p < 0.04).

The proportion of fever attributed to malaria was 37.8% (95% CI 32.9% – 42.7%). An age-specific pattern was observed with significant variations across different regions in the country. In general, among children less than 12 months of life, the proportion of fever attributed to malaria infection was 43.5% (95% CI 25.8% – 61.2%), in children aged between 12 and 59 months of age was 39.6% (95% CI 30.3% – 48.9%), and among children aged between 5 and 10 years old was 21.5% (95% CI 11.6% – 31.4%).

**Conclusion:**

This study confirms that malaria remains a major cause of febrile illness during childhood. It also defines the relation between parasite density and fever and how this varies with age and region. This may help guide case definition for clinical trials of preventive tools, as well as provide definitions that may improve the precision of measurement of the burden of disease.

## Background

In many malaria endemic regions, children acquire clinical immunity to malaria, and develop anti-parasitic mechanisms during the first few years of life consequent to repetitive exposures [1]. Thus, as children grow up, they develop an aptitude to lessen the density of malaria infection and to tolerate malaria parasites in the absence of fever [2]. It appears that the capability to tolerate malaria parasites increases with age, and is influenced by the intensity of malaria transmission. For this reason, clinical malaria is age-specific with regional and seasonal variations [3,4]. Additionally, in such areas, observation of asymptomatic malaria parasites carriers in the population is common, and detection of malaria parasites in a blood smear, does not certainly indicate a clinical malaria episode. Although the development of symptoms and/or signs of clinical malaria is complex and multi-factorial [5], in the recent years, emphasis on the relationship between fever risk and parasite density has been largely used as an entry point to define a clinical malaria episode [6].

The concept of "pyrogenic threshold" has been proposed for defining malaria episode in endemic areas [7-9]. The occurrence of fever cases due to other causes in the presence of parasitaemia, may well result in an over-diagnosis of clinical malaria. Thus, estimation of the proportion of fever cases attributable to malaria infection is crucial to establish a more concise definition of clinical malaria. Smith *et al *[6] proposed a methodology to estimate the attributable proportion of fever using a logistic regression of the fever on a monotonic function of the parasite density [10]. In addition, the sensitivity and specificity of specific cut-off values of parasite density can be estimated.

In Mozambique, malaria remains one of the main causes of febrile illness among children. Transmission is perennial with regional variations throughout the country. The incidence of clinical malaria established through weekly active case detection suggests that the risk of clinical malaria is highest between the age of one and three years when children experience an average of more than two episodes per year. Based on a continuous demographic surveillance system and verbal autopsies carried out in Manhiça district, the risk of clinical malaria drops sharply after the age of six [11].

Early diagnosis and prompt treatment of cases, especially in children represent the keystone strategy aiming at reducing the burden of malaria-related morbidity and mortality. However, in most rural areas, diagnosis of clinical malaria is rather presumptive based on fever or history of fever, though a positive blood film is required to confirm the diagnosis in areas where laboratory facilities exist.

This paper reports the results of a national malaria survey, carried out in Mozambique, in which the prevalence and the intensity of malaria infections, the establishment of malaria case definition and its relation to age strata across the country were determined.

## Methods

A detailed description of the methods used in this survey is presented in a companion paper [12]. Briefly, a total of 8,816 children aged below ten years of age, living in households selected from 24 districts chosen randomly in the country, were eligible for the survey. Oral informed consent was obtained and a questionnaire was completed by well-trained team members. Thick and thin smears for parasitological examination and parasite density estimation were obtained during the survey. Axillary temperatures were also recorded using digital thermometers. Overall prevalence of *Plasmodium falciparum *infections, fever, and malaria parasite infection associated with fever were estimated in each age group for each region and stratum.

Using the method proposed by Smith *et al *[6], the attributable proportion of fever due to malaria using a logistic regression of the fever on a monotonic function of the parasite density was estimated. The model used was *logit*(*π*_*i*_) = *α *+ *β*(*x*_*i*_)^τ^, and represents a logistic regression model where *π*_*I *_is the probability that observation *i *with parasite density *xi *is a fever case. *β*(*x*_*i*_)^*t *^corresponds to model type 3 described by Smith *et al *and is a monotonic function which is more flexible than the regression on *x*_*i *_or log(*x*_*i*_). The models were fitted using maximum likelihood. To constrain the parameter τ to be positive, the maximum likelihood were estimated for the log(τ). Confidence intervals for the estimated attributable fraction, the τ parameter, the sensitivity and specificity were obtained using the bootstrap methodology [13]. Analysis were made using Stata Statistical Software (Stata Corporation, College Station, Tx USA).

## Results

### Prevalence of fever and fever associated with malaria parasites

Overall, fever prevalence (axillary temperature ≥ 37.5°C) among children was 9.4% (766/8,816). Table 1, depicts the prevalence of parasite infection, fever and fever associated with parasite in different age groups. The prevalence of fever decreased rapidly with increasing age, peaking among children during the first 12 months of life [15.1% (206/1,517)]. The lowest fever prevalence of 5.9% (67/1,224) was recorded among children in the five to seven years of age group. Overall, the prevalence of fever associated with *P. falciparum *infections (asexual forms) accounted for 5.7% (498/8,816), but, among febrile children, 72.4% (554/766) were infected. The prevalence of malaria infection associated with fever peaked among children during the first year of life and thereafter decreased sharply with increasing age, and the differences among age groups were statistically significant (p < 0.001).

**Table 1:** Overall point prevalence of parasite, fever and fever associated with parasite in different age groups across the study area.

**Age group**	**Recorded** **N**	**Parasite** **(%)**	**Fever** **(%)**	**Parasite and fever** **(%)**
< 12 months	1,517	42.2	15.1	10.6

12 – 23 months	1,609	55.4	13.2	10.3

24 – 59 months	3,515	51.3	7.1	4.6

5 – < 7 years	1,224	48.1	5.9	3.2

7 – <10 years	951	39.3	5.3	3.3

Total	8,816	48.6	9.4	5.7

p-Value		< 0.001	< 0.001	< 0.001

The risk of being febrile increased with increasing parasite density, particularly from parasite density category equal or higher than 5,000 parasites/μl. High *P. falciparum *parasite densities were significantly associated with fever (p < 0.05) (Table 2). According to age group, the risk of fever increased during the first 12 months of life and thereafter decreased significantly with age (p < 0.0001). Across the study area there were significant regional variations (p < 0.001). The highest prevalence of malaria infection associated with fever was recorded in the northern region 16.6% (199/1,313), while in the southern region was recorded the lowest 9.9% (59/613). Although, there were variations across strata within regions, the differences observed were not statistically significant.

**Table 2:** Proportion of fever cases according to parasite density category

**Parasite Density (parasites/μl)**	**Fever Cases (%)**
0	6.9

1 – 499	7.2

500 – 4,999	10.4

5,000 – 49,000	21.3

≥ 50,000	42.1

p-Value	< 0.001

### The proportion of fever attributable to malaria infection

**Figure 1:**
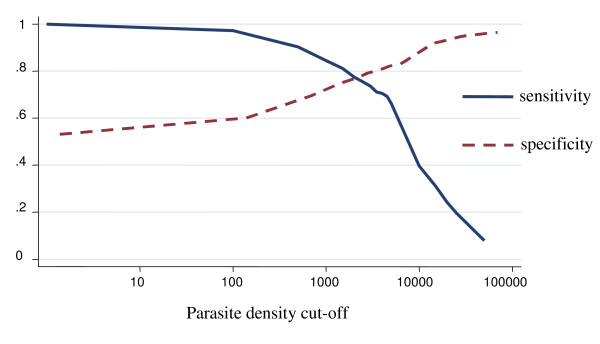
**Sensitivity and specificity curves and attributable proportion of clinical malaria among children in Mozambique**. Overall country-wide, children less than 10 years of age. Attributable proportion: 37.8%.

The model define malaria case as any case with at least one parasite, hence sensitivity of parasite density cut-off of one parasite or more will be always 100%. Table 3, illustrates the variations of the malaria attributable proportion of fever by age group across different epidemiological settings in the study area. Overall the proportion of fever attributable to malaria infection was 37.8% (95% CI 32.9% – 42.7%) (Figure 1). The sensitivity of parasite density cut-off of one parasite or more was 100%, and the specificity was 56.2 (95% CI 54.5% – 57.9%). Nevertheless for parasite density cut-off of 2,500 or more parasites, the sensitivity was 75.5% (95% CI 71.9% – 79.1%) and specificity was 83.3% (95% CI 82.4% – 84.3%). When adjusted for age group it dropped slightly to 37.5%. It decreased with increasing age, and the differences between age group categories were statistically significant (p < 0.05).

**Table 3:** Overall malaria attributable proportion of fever by age group across the study area.

**Age group**	**Fever cases** **N**	**Infected cases** **N**	**Attributable Proportion** **% (95% CI)**
< 12 months	206	641	43.5 (25.8 – 61.2)

12 – 29 months	439	2,556	39.6 (30.3 – 48.9)

5 – < 10 years	121	901	21.5 (11.6 – 31.4)

Total	766	4,098	37.8 (32.9 – 42.7)

p-Value		< 0.001	< 0.001

After adjusting for region, the fever proportion attributable to malaria was 36.5%, decreasing from the northern to the southern region, the differences were statistically significant (p < 0.05). When adjusted for stratum the attributable fraction of fever was 38.1%, but without significant variations across strata. When adjusted for age group and region the attributable fraction of fever dropped to 36.1% and the differences observed among regions and age groups were statistically significant (p < 0.05). When adjusted for age group, region and stratum there were no differences statistically significant across strata, however, across regions there were differences statistically significant (p < 0.05).

### Among children less than twelve months of age

**Figure 2:**
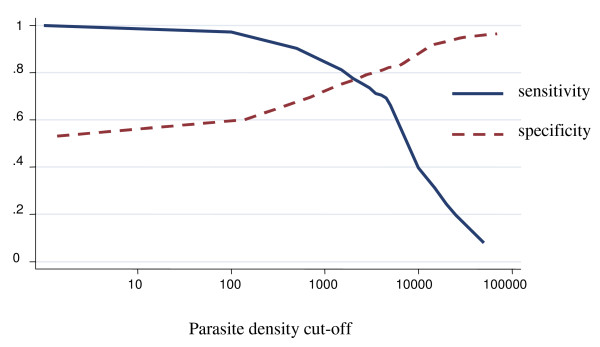
**Sensitivity and specificity curves and attributable proportion of clinical malaria among children in Mozambique**. Overall country-wide, children less than 12 months of age. Attributable proportion: 43.5%.

The proportion of fever attributable to malaria infection among children less than 12 months of life was 43.5% (95% CI 25.8% – 61.2%) (Figure 2). For parasite density cut-off of one or more parasites/μl, the specificity was 61.8% (95% CI 58.9% – 64.7%). For parasite density cut-off of 2,500 or more parasites/μl the sensitivity was 72.9% (95% CI 62.2% – 83.6%), and the specificity was 83.0 (95% CI 79.7% – 86.4%). After adjusting for region the attributable fraction dropped to 41.4%. Differences across regions were observed, but only for the central region the differences were statistically significant (p < 0.05). After adjusting for stratum it was 44.5%. It increased from the coastal strata to the highland strata, though, there were no differences statistically significant.

### Among children aged between 12 and 59 months

**Figure 3:**
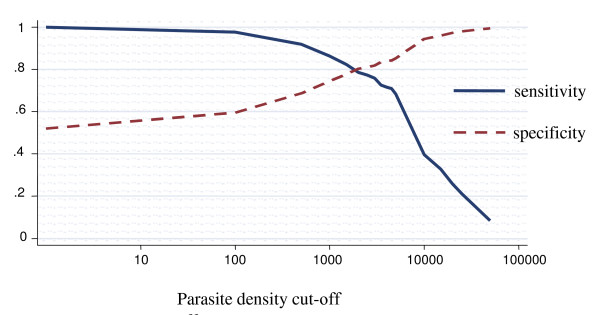
**Sensitivity and specificity curves and attributable proportion of clinical malaria among children in Mozambique**. Overall country-wide, children aged between 12 – 59 months. Attributable proportion: 39.6%

The proportion of fever attributable to malaria infection among children aged between 12 and 59 months of age was 39.6% (95% CI 30.3% – 48.9%) (Figure 3). For parasite density cut-off of one or more parasites/μl the specificity was 52.0% (95% CI 48.7% – 55.4%). For parasite density cut-off of 2,500 or more parasites/μl the sensitivity was 77.4% (95% CI 72.5% – 82.3%), and the specificity was 80.9 (95% CI 79.9% – 82.0%). After adjusting for region it dropped to 38.4%, decreasing form the northern to the southern region. The differences observed across regions were statistically significant (p < 0.05). When adjusted for strata it was 40.0%. It increased slightly from the coastal to the highland stratum. However, the differences were not statistically significant across strata. After adjusting for age group and region was 37.8%, increasing from the coastal to the highland stratum. It decreased from the northern to the southern region. There were differences statistically significant across regions. Nevertheless, after adjusting for age group and stratum there were no differences statistically significant across the strata.

### Among children above five years of age

**Figure 4:**
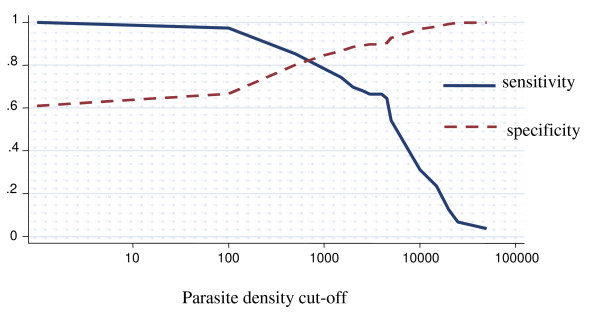
**Sensitivity and specificity curves and attributable proportion of clinical malaria among children in Mozambique**. Overall country-wide, children aged between 5 and 10 years. Attributable proportion: 21.5%.

The proportion of fever attributable to malaria infection among children aged between 5 and 10 years old was 21.5% (95% CI 11.6% – 31.4%) (Figure 4). For parasite density cut-off of one or more parasites/μl, the specificity was 61.1% (95% CI 56.5% – 65.6%). For parasite density cut-off of 2,500 or more parasites/μl the sensitivity was 68.2% (95% CI 45.5% – 90.9%), and the specificity was 89.2 (95% CI 85.1% – 93.3%). After adjusting for region it dropped to 20.2%, decreasing form the northern to the southern. The differences observed across regions were statistically significant (p < 0.05). When adjusted for stratum it was 21.2%, however, the differences observed across strata were not statistically significant.

### Variations across different epidemiological settings

**Figure 5:**
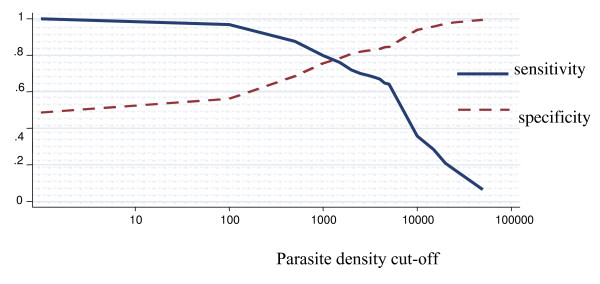
**Sensitivity and specificity curves and attributable proportion of clinical malaria among children in Mozambique**. Northern region. Attributable proportion: 37.0%.

**Figure 6:**
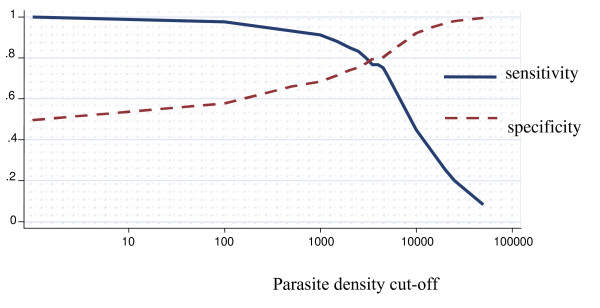
**Sensitivity and specificity curves and attributable proportion of clinical malaria among children in Mozambique**. Centre-Northern region. Attributable proportion: 48.2%.

**Figure 7:**
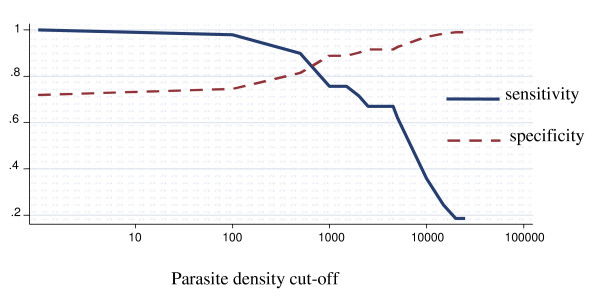
**Sensitivity and specificity curves and attributable proportion of clinical malaria among children in Mozambique**. Central region. Attributable proportion: 35.6%.

**Figure 8:**
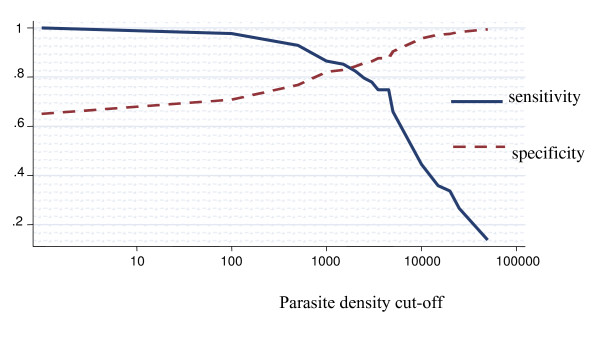
**Sensitivity and specificity curves and attributable proportion of clinical malaria among children in Mozambique**. Southern region. Attributable proportion: 31.1%.

Overall, malaria attributable proportion of fever showed variations throughout various regions in the country, decreasing from north-to-south. Within the northern region malaria attributable proportion of fever was 37.0% (Figure 5), the highest malaria attributable proportion of fever was recorded in the centre-northern region (Figure 6), while in the central and southern region were recorded the lowest malaria attributable proportion of fever of 35.5% (Figure 7) and 31.1% (Figure 8) respectively.

## Discussion

In this survey, the proportion of fever cases attributable to malaria was 37.8% (95% CI 32.9% – 42.7%), representing the proportion of febrile morbidity that would have been removed if malaria infections were eliminated among children in various settings in the study area. On the other hand, the results of this study highlighted the importance of other fever episodes not attributed to malaria infections in the study area.

In many malaria endemic areas, the burden of disease, a definition based on fever or reported episode of fever within the previous 48 hours in the presence of any level of parasitaemia, would result in an over-diagnosis of malaria cases. This study was conducted during high malaria transmission season, nevertheless, among febrile children approximately one third did not have malaria parasites. This finding has significant implications on the treatment policy, particularly in rural areas where in the absence of laboratorial diagnosis, all febrile cases would be considered as clinical malaria episodes. Moreover, even among febrile parasitized children, the proportion attributable to malaria infection was less than 40%.

The association between malaria infection and body temperature varies significantly among children. Despite, that the definition of clinical malaria have been related to fever episode and presence of parasites in the blood stream, in endemic-malaria areas, manifestations of clinical malaria have a wide spectrum [14] and the parasite density required to trigger fever differs significantly from one individual to another [4]. It was clear that all malaria cases had malaria parasites and fever, however, children with fever in the absence of malaria parasites are not necessarily non-malaria cases, since parasitaemia is a fluctuant variable and a child with malaria may have parasitaemia at undetectable levels at a given time.

In the study area, the majority of parasitized children were asymptomatic carriers, and not all fever episodes were associated with malaria parasites, hence very few fever episodes associated with asexual *P. falciparum *infections were observed. Additionally, the risk of fever among parasitized children was age-dependent, and increased with increasing mean parasite density.

In the study area, the overall proportion of fever cases attributable to the presence of malaria infection, when adjusted for age showed significant variations. Among younger children, it was 43.5% and decreased with increasing age to reach a low of 21.5% among older children. Therefore, the proportion of fever cases attributed to parasitaemia, the sensitivity and specificity of clinical malaria definition was age-specific. Consequently, among children exposed to malaria infection, the outcome or the risk of developing fever as a clinical manifestation of malaria infection, decreased with increasing age. These findings corroborate with results from other studies carried out in highly endemic areas [1,3,4].

Regional variations on the proportion of fever attributable to malaria infection among children have been reported from other community-based surveys in endemic-malaria areas [1], highlighting the changing patterns of the relationship between malaria parasites and the host [2,4]. Based on the analysis of the age specific sensitivity and specificity confidence intervals for the proportion of fever attributable to malaria, the sensitivity and specificity for the cut-off-points definition for one parasite/μl and 2,500 parasites/μl were obtained for different age groups. It was crucial to determine age specific sensitivity and specificity, otherwise lack of specificity would result in a biased estimate of case definition and lack of sensitivity would result in a loss of power. However, to obtain accurate estimates of the efficacy of one intervention in clinical studies, diagnostic specificity is more important thus age-specific cut-off and region specific should be considered.

The case definitions derived from this survey, have shown the relationship between parasite density and fever and how this varies with age and region. This may help guide case definition for clinical trials of preventive tools, as well as provide definitions that may improve the precision of burden of disease assessment.

This study confirms that malaria infection remains a major cause of febrile illness during childhood. However, other causes of fever should be considered in case management of febrile illnesses during childhood.

## Competing interests

The authors declare that they have no competing interests.

## Authors' contributions

SM made a substantial contribution on conception and design of the study, coordinated and supervised data collection in all regions, interpretation of data, performed all statistical analysis and wrote the manuscript. JA gave a major contribution on data interpretation and statistical analysis and helped to draft and revised the manuscript. AT helped to draft, gave contribution and critically revised the manuscript. PA gave a major contribution on conception and study design and helped to draft and critically revised the manuscript. All authors read and approved the final manuscript.
